# Change to hospitalist providers had a minimal influence on overall antibiotic use in a VA long-term care setting

**DOI:** 10.1017/ash.2023.334

**Published:** 2023-09-29

**Authors:** Taissa Bej, Brigid Wilson, Federico Perez, Robin Jump

## Abstract

**Background:** In long-term care settings, practice patterns among practitioners are stronger determinants of antibiotic use than resident characteristics. In July 2021, hospitalists from the acute medicine service replaced geriatricians and assumed the care of residents in a 110-bed community living center (CLC) at a large academic Veterans Affairs (VA) medical center. We assessed changes in antibiotic use associated with that change of practitioners to guide stewardship efforts. We hypothesized that antibiotic use in the CLC would shift, reflecting the practice pattern of practitioners accustomed to treating patients in acute-care settings. **Methods:** We conducted a retrospective cohort study from July 1, 2020, through June 30, 2022, 1 year before and after the change of practitioners on July 1, 2021. We assessed resident characteristics and the following metrics of antibiotic use at monthly intervals: days of therapy (DOT) per 1,000 bed days of care (BDOC), antibiotic starts per 1,000 BDOC, and mean length of therapy (LOT) in days. We also compared the DOT per 1,000 BDOC for various antibiotics, in groups and individually. **Results:** In the years before and after the change of practitioners on July 1, 2021, the characteristics of CLC residents were comparable. Before and after July 1, 2021, monthly DOT per 1,000 BDOC (Fig. 1A), antibiotic starts per 1,000 BDOC, and mean LOT (Fig. 1B) were similar. After July 1, 2021, the use of fluoroquinolones decreased (14.31 vs 5.83 DOT per 1,000 BDOC; *P* < .01), and variations in anti-MRSA, narrow-spectrum, and broad-spectrum hospital agents were small, whereas the use of broad-spectrum community agents increased (29.42 vs 47.81 DOT per 1,000 BDOC; *P* < .01) (Fig. 2A). Within this group, there was increased use of doxycycline (7.42 vs 19.13 DOT per 1,000 BDOC; *P* < .01), ertapenem (2.03 vs 4.58 DOT per 1,000 BDOC; *P* < .01), and, modestly, azithromycin (0.40 vs 1.80 DOT per 1,000 BDOC) (Fig. 2B). **Conclusions:** The overall use of antibiotics, as measured by DOT, antibiotic starts, and LOT did not change after hospitalists assumed care of CLC residents. However, a notable decrease was observed in the use of fluoroquinolones, and an increase was observed in the use of doxycycline and ertapenem. Stewardship that is tailored to the type of provider and incorporates their practice patterns is needed to reinforce the prudent use of antibiotics.

**Figure:**
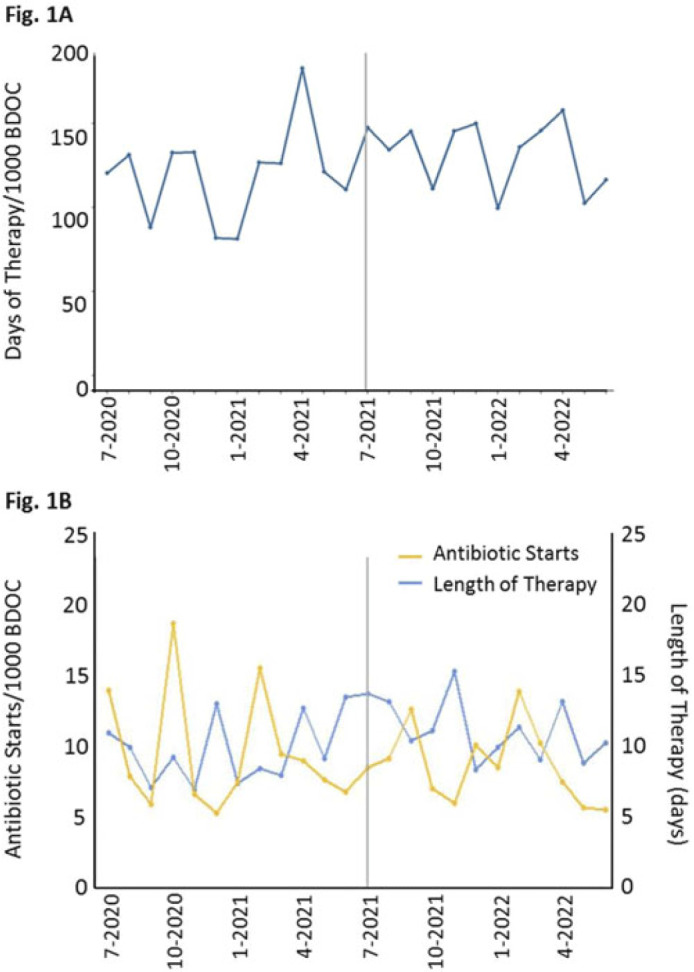

**Figure:**
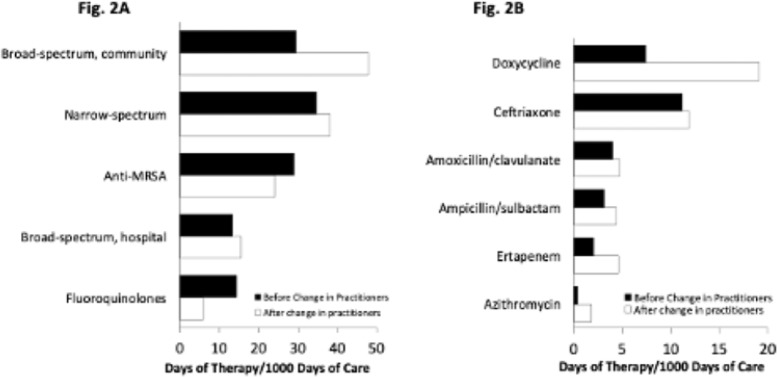

**Disclosures:** None

